# Meta-analysis and meta-modelling for diagnostic problems

**DOI:** 10.1186/1471-2288-14-56

**Published:** 2014-04-24

**Authors:** Suphada Charoensawat, Walailuck Böhning, Dankmar Böhning, Heinz Holling

**Affiliations:** 1 Health Sciences Program Udon Thani Rajabhat University, Udon Thani, Thailand; 2 Statistics and Quantitative Methods, Faculty of Psychology and Sport Science, University of Münster, Münster, Germany; 3 Southampton Statistical Sciences Research Institute, Mathematics and Medical Statistics, University of Southampton, Southampton SO17 1BJ, UK

**Keywords:** Diagnostic accuracy, Mixed modelling, Random effects modelling, Cut-off value modelling, SROC modelling

## Abstract

**Background:**

A proportional hazards measure is suggested in the context of analyzing SROC curves that arise in the meta–analysis of diagnostic studies. The measure can be motivated as a special model: the Lehmann model for ROC curves. The Lehmann model involves study–specific sensitivities and specificities and a diagnostic accuracy parameter which connects the two.

**Methods:**

A study–specific model is estimated for each study, and the resulting study-specific estimate of diagnostic accuracy is taken as an outcome measure for a mixed model with a random study effect and other study-level covariates as fixed effects. The variance component model becomes estimable by deriving within-study variances, depending on the outcome measure of choice. In contrast to existing approaches – usually of bivariate nature for the outcome measures – the suggested approach is univariate and, hence, allows easily the application of conventional mixed modelling.

**Results:**

Some simple modifications in the SAS procedure proc mixed allow the fitting of mixed models for meta-analytic data from diagnostic studies. The methodology is illustrated with several meta–analytic diagnostic data sets, including a meta–analysis of the Mini–Mental State Examination as a diagnostic device for dementia and mild cognitive impairment.

**Conclusions:**

The proposed methodology allows us to embed the meta-analysis of diagnostic studies into the well–developed area of mixed modelling. Different outcome measures, specifically from the perspective of whether a local or a global measure of diagnostic accuracy should be applied, are discussed as well. In particular, variation in cut-off value is discussed together with recommendations on choosing the best cut-off value. We also show how this problem can be addressed with the proposed methodology.

## Background

We are interested in the following setting occurring in the field of meta-analysis of diagnostic studies (Hasselblad and Hedges [1]; Sutton *et al.*[2]; Deeks [3]; Schulze *et al.*[4]): a variety of diagnostic studies are available providing estimates of the diagnostic measures of specificity *q*=*P*(*T*=0|*D*=0) as ${\hat{q}}_{i}=x_{i}/n_{i}$ and of sensitivity *p*=*P*(*T*=1|*D*=1) as ${\hat{p}}_{i}=y_{i}/m_{i}$, where *D*=1 and *D*=0 denote presence or absence of disease, respectively, and *T*=1 or *T*=0 denote positivity or negativity of the diagnostic test, respectively, *x*_*i*_ are the number of observed true-negatives out of *n*_*i*_ healthy individuals, and *y*_*i*_ are the number of observed true-positives out of *m*_*i*_ diseased individuals, for *i*=1,…,*k*, *k* being the number of studies. For more details on the statistical modelling of the diagnostic data from a single study, see Pepe [5,6]. For a more detailed introduction to meta–analysis of diagnostic studies, see Holling *et al.*[7]. In the following, we will look at several examples – mainly from medicine and psychology – for this special meta-analytic situation. In principle, however, applications could occur in all areas in which meta-analytic data is encountered; Swets [8] considers mainly psychological applications, but also mentions cases from engineering (quality control), manufacturing (failing parts in planes), metereology (correctness of weather predictions), information science (correctness of information retrieval), or criminology (correctness of lie detection test). We illustrate the special meta-analytic situation mentioned above with a meta-analysis on a diagnostic test on heart failure (see also Holling *et al.*[7]).

*Example 1: Meta-Analysis of diagnostic accuracy of Brain Natriuretic Peptides (BNP) for heart failure.* Doust *et al.*[9] provide a meta-analysis on the diagnostic accuracy of the brain natriuretic peptides (BNP) procedure as a diagnostic test for heart failure. According to the authors, diagnosis of heart failure is difficult, with both overdiagnosis and underdiagnosis occurring. The meta-analysis considers a range of diagnostic studies that use different reference standards (where a reference standard defines the presence or absence of disease). Here we only consider the eight studies (see Table 1) using the left ventricular ejection fraction of 40% or less as reference standard.

**Table 1:** Meta-analysis of of diagnostic accuracy of brain natriuretic peptides (BNP) for heart failure using the left ventricular ejection fraction of 40% or less as reference standard

	**Diseased**		**Healthy**		
**Study** ***i***	***y***_***i***_**(TP)**	***m***_***i***_**−*y***_***i***_**(FN)**	***x***_***i***_**(TN)**	***n***_***i***_**−*x***_***i***_**(FP)**	***n***_***i***_**+** ***m***_***i***_
Bettencourt 2000	29	7	46	19	101
Choy 1994	34	6	22	13	75
Valli 2001	49	9	78	17	153
Vasan 2002a	4	6	1612	85	1707
Vasan 2002b	20	40	1339	71	1470
Hutcheon 2002	29	2	102	166	299
Landray 2000	26	14	75	11	126
Smith 2000	11	1	93	50	155

*The cut–off value problem.* A separate meta–analysis of sensitivity and specificity using the meta–analytic tools for independent binomial samples is problematic when the underlying diagnostic test utilizes a continuous or ordered categorical scale and different cut–off values have been used in different diagnostic studies. A simple variation of the cut–off value from study to study might lead to quite different values of sensitivity and specificity without any actual change in the diagnostic accuracy of the underlying test.

**Figure 1:**
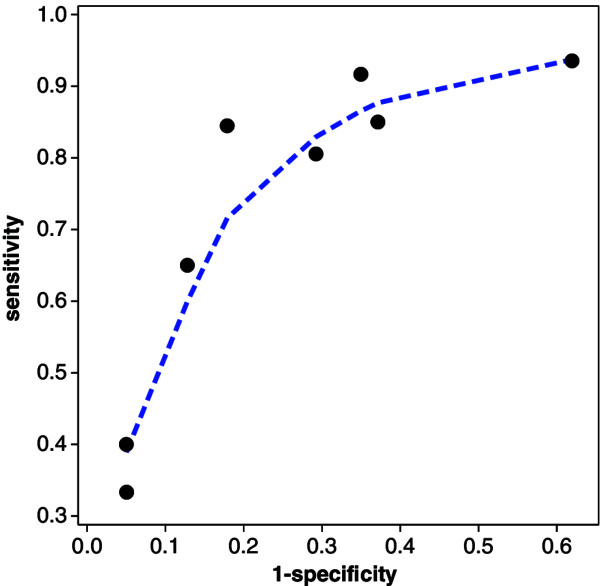
SROC diagram for BNP and heart failure: circles are the observed pairs of false positive rate and sensitivity, dashed curve is lowess smoother.

*SROC curve.* Due to this comparability problem for sensitivity and specificity, interest is usually focussed on the *summary receiver operating characteristic* (SROC) curve consisting of the pairs (1−*q*(*t*),*p*(*t*)) where *q*(*t*)= *P*(*T*<*t*|*D*=0) and *p*(*t*)=*P*(*T*≥*t*|*D*=1) for a continuous test *T* with potential value *t*. For a given study *i*, *i*=1,⋯,*k*, with potentially unknown cut–off value *t*_*i*_, the pairs (1−*q*(*t*_*i*_),*p*(*t*_*i*_)) can be estimated by $\left(1-{\hat{q}}_{i},{\hat{p}}_{i})=(1-x_{i}/n_{i},y_{i}/m_{i}\right)$ for *i*=1,…,*k*. The SROC curve accommodates the cut–off value problem. Different pairs could have quite different values of specificity and sensitivity, but still reflect identical diagnostic accuracy. The SROC diagram for the meta–analysis on BNP and heart failure is given in Figure 1.

Clearly, there is a wide range of values for specificity and sensitivity. Nevertheless, as Figure 1 shows, the possibility that the pairs might stem from a common SROC curve (as given by the dashed curve in Figure 1) cannot be discarded. Since the SROC approach accommodates the cut-off value problem, it is commonly preferred to summary measures like the Youden index [10] or the diagnostic odds ratio [11]. In the following, we focus our analysis on the SROC curve.

*Background of SROC modelling.* SROC modelling has received considerable attention in the field and experienced several developments. An early model was suggested by Littenberg and Moses [12], [13] and has been used in practice frequently; Deeks [3] discusses its prominent role in modeling meta-analytic diagnostic study accuracy. Littenberg and Moses [13] suggest fitting *D*=*α*+*βS*, where $D=\log\text{DOR}=\log\frac{p}{1-p}-\log\frac{1-q}{q}$ is the *log-diagnostic odds ratio* and $S=\log\frac{p}{1-p}+\log\frac{1-q}{q}$ is a measure for a potential threshold effect. After *α* and *β* have been estimated from the data, the SROC-curve (*p* vs. 1−*q*) is reconstructed from the estimated values of *α* and *β*. The parameter *α* is interpreted as the *summary log-DOR*, which is adjusted by means of *S* for potential *cut-off value effect*.

A two–level approach has been suggested by Rutter and Gatsonis [14], which is typically given in the following notational form (Walter and Macaskill [15]): let *Z*_*i* *j*_∼*Bi*(*n*_*i* *j*_,*π*_*i* *j*_), where *Z*_*i* *j*_ is the number of test-positives in study *i* for arm *j* (*j*=1 is diseased, *j*=2 is non-diseased), *n*_*i* *j*_ is the size of arm *j* in study *i* and *π*_*i*1_ is the sensitivity, *π*_*i*2_ is the false positive rate; the model is $\log\frac{\pi_{\text{ij}}}{1-\pi_{\text{ij}}}=(\theta_{i}+\alpha_{i}{\text{DS}}_{\text{ij}})\exp(-\beta {\text{DS}}_{\text{ij}}),$ where *θ*_*i*_ is an implicit threshold parameter for study *i*, *α*_*i*_ is the diagnostic accuracy parameter in study *i*, and *DS*_*i* *j*_ represents a binary variable for the disease status. The parameter *β* allows for an association between test accuracy and test threshold. When *β*=0, *α*_*i*_ is estimated by *D*_*i*_ and *θ*_*i*_ is estimated by *S*_*i*_/2, where *D*_*i*_ and *S*_*i*_ are as for the Littenberg–Moses model. Furthermore, to account for between-study variation, a random effect is assumed for $\theta_{i}∼N(\Theta ,\tau_{\theta }^{2})$ and $\alpha_{i}∼N(\Lambda ,\tau_{\alpha }^{2})$, with *θ*_*i*_ and *α*_*i*_ being independent. As an alternative, a bivariate normal random-effects meta–analysis has been suggested by van Houwelingen *et al.*[16]; see also Reitsma *et al.*[17] and Arends *et al.*[18]. Harbord *et al.*[19] show that these models are closely related.

*Paper overview.* In the following, we propose a specific model, called the Lehmann model, which we believe is very attractive for the analysis of SROC curves. The model involves study–specific sensitivities and specificities and a diagnostic accuracy parameter which connects the two. The Lehmann model achieves flexibility by allowing the diagnostic accuracy parameter to become a random effect. In this it is similar to the Rutter-Gatsonis model, but differs in that it retains univariate dimensionality in its outcome measure and, hence, allows a mixed model approach in a more conventional way. In section “The proportional hazards measure”, the proportional hazards measure is motivated as a specific form of SROC curve modelling and is compared to other approaches. Section “A mixed model approach” introduces the specific mixed model in which the log proportional hazards measure forms the outcome measure, the study factor is a normally distributed random effect (to cope with unobserved heterogeneity), and other observed covariates (such as gold standard or diagnostic test variation) are considered as fixed effects in the mixed model. Section “Results” considers various applications including a meta-analysis of the Mini-Mental State Examination to diagnose dementia or mild cognitive impairment. It also provides SAS-code for a simple execution of the suggested approach. In section “Discussion”, the choice of outcome is discussed and the difference between global and local diagnostic accuracy measures highlighted. This is particularly of interest if observed cut-off value variation occurs in the meta-analysis and needs to be assessed. Here a local criterion of diagnostic accuracy appears more appropriate. The paper ends with some brief conclusions and discussion in section “Conclusions”.

## Methods

### The proportional hazards measure

**Figure 2:**
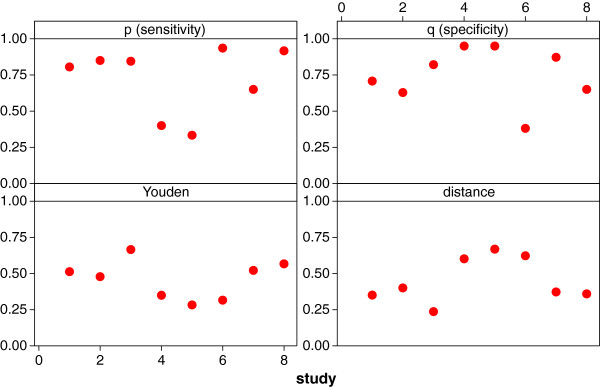
Index plots for sensitivity, specificity, Youden index, and Euclidean distance showing the wide variability of these measures for the data of the meta-analysis of BNP and heart failure.

Numerous summary measures for a pair of specificity and sensitivity have been suggested: we mention here the Youden index, *J*_*i*_=*p*_*i*_+*q*_*i*_−1 [10], and the squared Euclidean distance to the upper left corner in the SROC diagram, *E*_*i*_=(1−*p*_*i*_)^2^+(1−*q*_*i*_)^2^. [A review of summary measures is given in Liu [20].] Using an average over any of these measures might be problematic: not only might sensitivities and specificities be heterogeneous, this might also be true for the associated summary measures such as the Youden index or the Euclidean distance (as demonstrated by Figure 2 using the data of the meta-analysis of BNP and heart failure).

**Figure 3:**
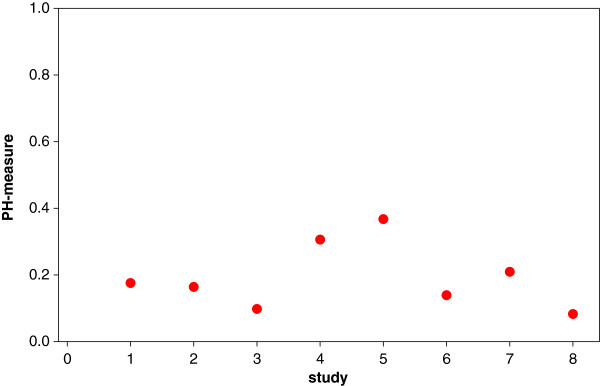
Index plots for the PH measures for the data of the meta-analysis of BNP and heart failure.

We suggest using the measure $\theta =\frac{\log p}{\log(1-q)}$, which relates the log-sensitivity to the log-false positive rate; we call it the *proportional hazards (PH)* measure. In Figure 3 we see that this measure shows a reduced variability for the meta-analysis of BNP and heart failure, making it more suitable as an overall measure in the meta-analysis of diagnostic studies or diagnostic problems. While the measure appears to be like any other summary measure of the pair sensitivity and specificity, it has a specific SROC-modelling background and motivation. We have mentioned previously the cut-off value problem: observed heterogeneity might be induced by cut-off value variation which could lead to different sensitivities and specificities – despite the accuracy of the diagnostic test itself not having changed – and might also lead to an induced heterogeneity in the summary measure. Hence, it is unclear whether the observed heterogeneity is due to heterogeneity in the diagnostic accuracy (authentic heterogeneity) or whether it has occurred due to cut-off value variation (artificial heterogeneity). This second form of heterogeneity can also occur when the background population changes with the study.

One of the features of the SROC approach is that it incorporates the cut-off value variation in a natural way; hence a measure modelling an ROC curve is favorable. We suggest the PH measure based upon the Lehman family in the following way:

(1) $$p={\left(1-q\right)}^{\theta }.$$

This model was suggested by Le [21] for the ROC curve. It is an appropriate model since, for feasible *q*, (1−*q*)^*θ*^ is also feasible as long as *θ* is positive. Note that (1) is defined for all values of *p*∈[0,1] and *q*∈ [ 0,1] whereas $\theta =\frac{\log p}{\log(1-q)}$ is only defined for *p*∈(0,1) and *q*∈(0,1). Population values of sensitivity and specificity of 1 are rarely realistic, although observed values of 1 for sensitivity and specificity do occur in samples. This can be coped with by using an appropriate smoothing constant such as estimating specificity as (*n*_*i*_−1)/*n*_*i*_ when *x*_*i*_=*n*_*i*_ and sensitivity as (*m*_*i*_−1)/*m*_*i*_ if *y*_*i*_=*m*_*i*_.

**Figure 4:**
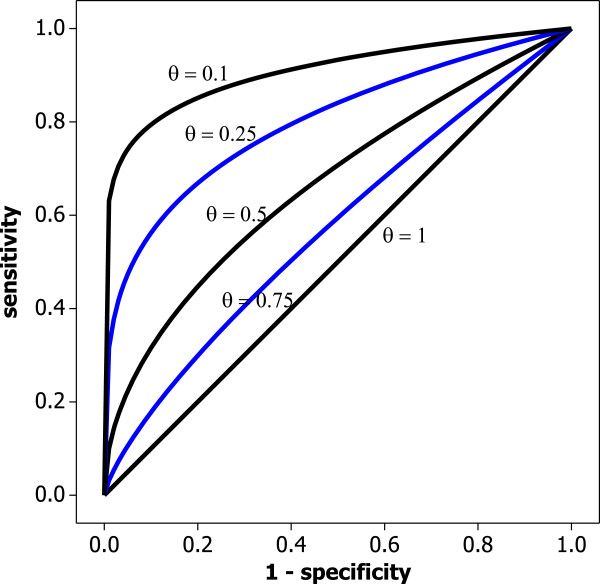
**Some examples of the proportional hazards model for various values of** ***θ*.**

In Figure 4 we see a number of examples of the proportional hazards family. It becomes clear now why *θ* is called the proportional hazards measure. By taking logarithms on both sides of (1) we achieve

**Figure 5:**
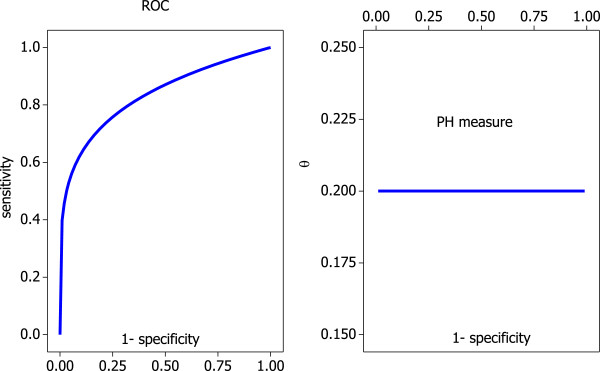
Proportional hazards model and associated PH measure.

(2) θ=logp(t)/log[1−q(t)],

meaning if model (1) holds, the ratio of log-sensitivity to log-false positive rate is constant across the range of possible cut-off value choices *t*. Hence the name proportional hazards model, which was suggested in a paper by Le [21] and used again in Gönen and Heller [22]. The idea of representing an entire ROC curve in a *single* measure is illustrated in Figure 5. While sensitivity and specificity vary over the entire interval (0,1), the value of *θ* remains constant. Hence, log-sensitivity is *proportional* to the log-false positive rate. This assumption is similar to an assumption used for a model in survival analysis, where it is assumed that the hazard rate of interest is proportional to the baseline hazard rate; this might have motivated the choice of name used by Le [21] and Gönen and Heller [22] in this context.

**Figure 6:**
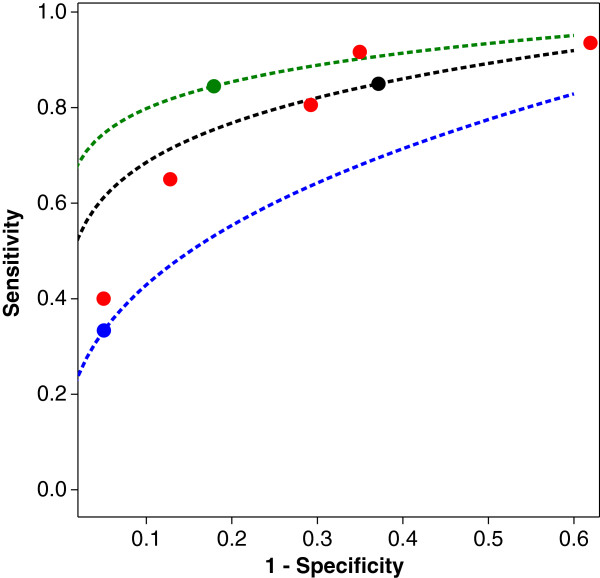
**Meta-analysis of BNP and heart failure: each study is represented by its own PH model (**1**) – illustrated for 3 studies.**

However, it is not our intention to make the assumption that an entire SROC curve can be represented by model (1); the explanations above are instead meant as a motivation that the PH-measure is not just another summary measure, but can be derived from a ROC modelling perspective. We envisage that each study, with associated pair of sensitivity and specificity, can be represented by a specific PH-model, as illustrated in Figure 6.

We see indeed that each pair of sensitivity and specificity can be associated with its own ROC curve provided by

(3) $$p={\left(1-q\right)}^{{\hat{\theta }}_{i}}$$

where ${\hat{\theta }}_{i}=\log{\hat{p}}_{i}/\log\;[\;1-{\hat{q}}_{i}]$, so that the curve (3) passes exactly through the point $\left(1-{\hat{q}}_{i},{\hat{p}}_{i}\right)$.

*Comparison to other approaches.* It remains to be seen how appropriate the suggested proportional hazards model is and how it compares to other existing approaches. We emphasize that in our situation we have assumed that there is only *one* pair of sensitivity and false positive rate $\left({\hat{p}}_{i},1-{\hat{q}}_{i}\right)$ per study *i*. Situations where several pairs per study are observed (such as in Aertgeerts *et al.*[23]) are rare. Hence, on the log-scale for sensitivity and false-positive rate, we are not able to identify any straight line model *within a study* with *more than one* parameter, since this would require at least two pairs of sensitivity and specificity per study; see also Rücker and Schumacher [24,25]. However, any one-parameter straight line model, such as the proposed proportional hazards model, is estimable within each study, although within-model diagnostics is limited since we are fitting the full within study model. Given that sample sizes within each diagnostic study are typically at least moderately large it seems reasonable to assume a bivariate normal distribution for $\log\hat{p}$ and $\log(1-\hat{q})$ with means log*p* and log(1−*q*) as well as variances $\sigma_{p}^{2}$ and $\sigma_{q}^{2}$, respectively, and covariance *σ* with correlation *ρ*=*σ*/(*σ*_*p*_*σ*_*q*_). This is very similar to the assumptions in the approach taken by Reitsma *et al.*[17] (see also Harbord *et al.*[19]), with the difference that we are using the log-transformation whereas in Reitsma *et al.*[17] logit-transformations are applied. Then, it is a well-known result that the mean of the random variable $\log\hat{p}$ (having unconditional mean log*p*) conditional upon the value of the random variable $\log(1-\hat{q})$ (having unconditional mean log(1−*q*)) is provided as

(4) $$E(\log\hat{p}|\log(1-\hat{q}))=\log p+\rho \frac{\sigma_{p}}{\sigma_{q}}[\;\log(1-\hat{q})-\log(1-q)],$$

which can be written as $\alpha +\theta [\;\log(1-\hat{q})]$ where *α*= log(*p*)−*θ* log(1−*q*) and $\theta =\rho \frac{\sigma_{p}}{\sigma_{q}}$. This is an *important* result since it means that, in the log-space, sensitivity and false–positive rate are linearly related. Furthermore, if *α* is zero, the proportional hazards model arises.

The question then arises why not work with a straight line model

(5) $$\log p_{\left|\log(1-q\right)}=\alpha +\theta \log(1-q).$$

The answer is that such a model is *not identifiable* since we have only one pair of sensitivity and specificity observed in each study and it is not possible to uniquely determine a straight line by just one pair of observations since there are infinitely many possible lines passing through a given point in the log*p* – log(1−*q*) space. However, the proportional hazards model as a slope-only model *is* identifiable and it is more plausible than other identifiable models such as the intercept–only model. Clearly, a logistic-transformation would be more consistent with the existing literature [14,15] than the log-transformation. However, both models would give a perfect fit (within each study) since there are no degrees of freedom left for testing the model fit. The situation changes when there are repeated observations of sensitivity and specificity *per study* available. However, these meta-analyses with repeated observations of sensitivity and specificity according to cut-off value variation are extremely rare.

### A mixed model approach

With the motivation of the previous sections in mind, we assume that *k* diagnostic studies are available with diagnostic accuracies ${\hat{\theta }}_{1},\cdots \;,{\hat{\theta }}_{k}$ where

(6) $${\hat{\theta }}_{i}=\frac{\log{\hat{p}}_{i}}{\log(1-{\hat{q}}_{i})}.$$

We assume the following linear mixed model for $\log{\hat{\theta }}_{i}$:

(7) $$\log{\hat{\theta }}_{i}={\beta^{T}x}_{i}+\delta_{i}+\varepsilon_{i}$$

where **x**_*i*_ is a known covariate vector in study *i*, *δ*_*i*_ is a normally distributed random effect *δ*_*i*_∼*N*(0,*τ*^2^) with *τ*^2^ being an unknown variance parameter, and $\varepsilon_{i}∼N(0,\sigma_{i}^{2})$ is a normally distributed random error with variance $\sigma_{i}^{2}$ known from the *i*−th study.

There are several noteworthy points about the mixed model (7). The response is measured on the log-scale, where the transformation improves the normal approximation and also brings the diagnostic accuracy into a well-known link function family: the complementary log-log function. The difference of the probability for a positive test in the groups with and without the condition is measured on the complementary log-log scale. The fixed effect part involves a covariate vector **x** which could contain information on study level such as gold standard variation, diagnostic test variation, or sample size information. It should be noted that there are two variance components, *τ*^2^ and $\sigma_{i}^{2}$. It is important to have information on the second variance component. If the second component is unknown, even under the assumption of homogeneity $\sigma_{1}^{2}=\cdots =\sigma_{k}^{2}$, the variance component model would *not* be identifiable. Hence, we need to devote some effort to derive expressions for the within study variances; this can be accomplished using the *δ*−method as discussed in the next section.

*Within study variance.* Let us consider (ignoring the study index *i* for the sake of simplicity)

(8) $$\log\hat{\theta }=\log(-\log\hat{p})-\log\;[\;-\log(1-\hat{q})]$$

and apply the *δ*−method. Recall that the variance *VarT*(*X*) of a transformed random variable *T*(*X*) can be approximated as [ *T*^′^(*E*(*X*))]^2^*Var*(*X*) assuming that the variance *Var*(*X*) of *X* is known. Applying this *δ*−method twice gives

(9) $$\text{Var}\log(-\log\hat{p})\approx \frac{\hat{p}(1-\hat{p})/m}{{\hat{p}}^{2}{\left(\log\hat{p}\right)}^{2}}$$

and

(10) $$\text{Var}\log(-\log(1-\hat{q}))\approx \frac{\hat{q}(1-\hat{q})/n}{{\left(1-\hat{q}\right)}^{2}{\left(\log(1-\hat{q})\right)}^{2}}$$

so that the within study variance for the *i*-th study is provided as

(11) $$\sigma_{i}^{2}=\frac{m_{i}-y_{i}}{m_{i}y_{i}{\left(\log y_{i}/m_{i}\right)}^{2}}+\frac{x_{i}}{n_{i}(n_{i}-x_{i}){\left(\log(1-x_{i}/n_{i})\right)}^{2}}.$$

We acknowledge that the above are estimates of the variances of the diagnostic accuracy estimates, but are used as if they were the true variances.

*Some important cases.* If there are no further covariates, *two* important models are easily identified as special cases of (7). One is the *fixed* effects model

(12) $$\log{\hat{\theta }}_{i}=\beta_{0}+\varepsilon_{i}$$

and the other is the *random* effects model

(13) $$\log{\hat{\theta }}_{i}=\beta_{0}+\delta_{i}+\varepsilon_{i}$$

which have gained some popularity in the meta-analytic literature.

## Results

### Case study on MMSE and dementia

**Figure 7:**
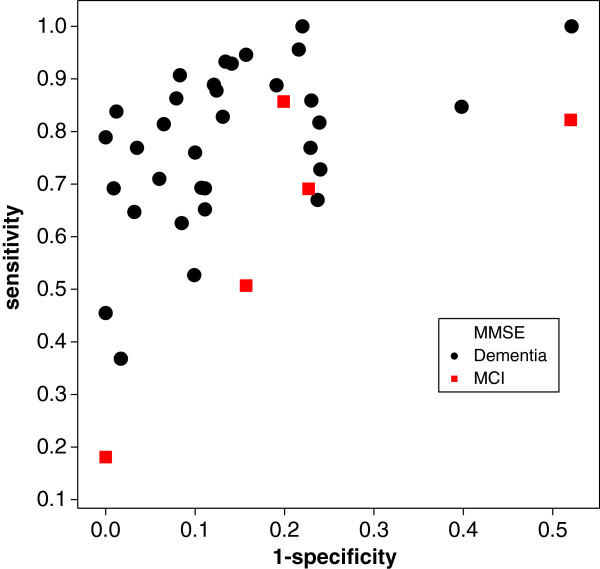
SROC diagram for the meta–analysis of MMSE and dementia or MCI as reference standard.

We illustrate the approach with an example and revisit a meta–analysis by Mitchell [26] on the diagnostic accuracy of the mini-mental state examination (MMSE) as a diagnostic test for the detection of dementia and, more recently, mild cognitive impairment (MCI). In this meta–analysis 38 studies were included and the entire data are reproduced in Table 2. We are interested in the question: is there a difference in diagnostic accuracy of the MMSE in the detection of dementia and MCI, as Figure 7 suggests.

**Table 2:** Meta-analysis of the diagnostic accuracy of the mini-mental state examination (MMSE) and dementia or mild cognitive impairment (MCI) as reference standard; TP = true positives, FN = false negatives, FP = false positives, TN = true negatives

**Study**	**Condition**	**TP**	**FN**	**FP**	**TN**
1	Dementia	65	3	240	870
2	Dementia	117	12	10	110
3	Dementia	48	19	63	989
4	Dementia	134	8	28	152
5	Dementia	24	5	44	292
6	Dementia	67	15	48	153
7	Dementia	64	17	1	71
8	Dementia	281	64	20	286
9	Dementia	13	1	44	286
10	Dementia	262	20	29	177
11	Dementia	143	18	29	123
12	Dementia	183	33	33	51
13	Dementia	22	1	152	140
14	Dementia	112	1	590	2091
15	Dementia	152	81	126	1009
16	Dementia	29	26	26	236
17	Dementia	31	6	3	247
18	Dementia	10	3	12	333
19	Dementia	707	88	1438	10447
20	Dementia	181	108	17	184
21	Dementia	59	29	23	74
22	Dementia	74	23	16	143
23	Dementia	27	12	26	209
24	Dementia	40	6	75	528
25	Dementia	317	52	173	578
26	Dementia	387	116	16	54
27	Dementia	118	65	1	44
28	Dementia	44	7	34	396
29	Dementia	123	46	98	309
30	Dementia	25	43	3	171
31	Dementia	73	32	2	225
32	Dementia	37	45	1	440
33	Dementia	78	34	45	376
34	MCI	72	12	53	214
35	MCI	106	23	410	379
36	MCI	37	36	22	118
37	MCI	67	30	22	75
38	MCI	17	77	1	90

We use proc mixed from the SAS software, version 9.2 for Windows [27], for the analysis (see also Table 3). The values of the dependent variable $\log{\hat{\theta }}_{i}$ are easily constructed from Table 2. We are interested to see if there are differences in accuracy for diagnosing MCI compared to diagnosing dementia. Hence we have constructed a covariate condition which takes the value 1 if the study concerns MCI as condition and 0 if the study is on dementia. Since we have fixed within-study variances, we need to tell proc mixed to incorporate this appropriately; this can be accomplished by using a weight, $w_{i}=1/\sigma_{i}^{2}$. The random option induces a random effect (here study) with associated variance component *τ*^2^, which is estimated. However, SAS proc mixed will automatically fit a within-study variance component (on top of the provided variances). To circumvent this mechanism, the option parms (1) (1) /hold=2 is used where the term hold=2 fixes the second variance component, corresponding to the within-study variance multiplier, to one. Note that the random effect modelling between-study variation is described by a free variance parameter, *τ*^2^. For this a starting value needs to be given: we have *τ*^2^=1, although other choices are possible, e.g. *τ*^2^=0, corresponding to the case of no heterogeneity between studies.

**Table 3:** **SAS proc **mixed** adapted for meta–analysis of diagnostic accuracy study data**

**SAS statement**	**Explanation**
proc mixed data=MMSE method=ml covtest;	procedure mixed of SAS, data contains the data file, method
	specifies estimation
class study condition;	defines the categorical variables used
model logtheta = condition/s;	defines the model: LHS outcome, RHS covariates used
weight w;	w contains inverse variance as weight
random study(condition);	factor study nested in condition
parms (1) (1)/hold=2;	specifies starting values, hold=2 fixes the residual variance component
run;	executes the program

The results of the analysis are provided in Table 4. It can be seen that there is a significant effect of condition (dementia/MCI) on the diagnostic accuracy, with diagnostic accuracy being significantly higher in studies with patients having dementia in comparison to the diagnostic accuracy in studies with patients having mild cognitive impairment. Nevertheless, not all heterogeneity is explained by this covariate as the random effect (study effect) still remains significant, as the bottom part of Table 4 shows.

**Table 4:** Analysis of effects for the meta-analysis of the diagnostic accuracy of the mini-mental state examination (MMSE) and dementia or mild cognitive impairment (MCI) as reference standard

**Effect**	**Parameter**	**SE**	**Z-value**
	**estimate**		
fixed			
Intercept	-2.2878	0.1208	-18.94
condition	0.8605	0.3187	2.70
random			
*τ*^2^ (study)	0.3078	0.1049	2.90

The inference is based here on a procedure called the Wald test. The estimated parameter value is divided by its estimated standard error, and the result is given in column four in Table 4. The likelihood ratio test may be considered as an alternative. It is defined as two times the difference of the log-likelihood including the effect of interest and the log-likelihood not including the effect of interest. For the effect of condition in Table 4, we find a value of 6.8 for the likelihood-ratio test. The Wald test is asymptotically standard normal under the null-hypothesis of absence of effect, whereas the likelihood ratio test statistic is asymptotically chi-squared distributed with degrees of freedom equal to the number of parameters associated with the effect considered (in this case one). It is well-known that the likelihood ratio test is more powerful. Here, both tests provide similar p-values, with 0.0091 for the likelihood ratio test and 0.0069 for the Wald test; this confirms the significance of the effect (dementia/MCI) on the diagnostic accuracy.

It is trivial to construct the associated SROC curves from Table 4. We find

$$\begin{array}{lll} \text{for dementia:}\;p & ={\left(1-q\right)}^{\exp(-2.2878)},\; & \;\\ \;\text{for MCI:}p & ={\left(1-q\right)}^{\exp(-2.2878+0.8605)}.\; & \; \end{array}$$

Note that the likelihood ratio test as well as the Wald test need modification in situations where the null hypothesis is part of the boundary of the alternative such as when testing H _0_:*τ*^2^=0. In this case, the asymptotic null distribution of the likelihood ratio test statistic is no longer *χ*^2^ with 1 df but rather a mixture of a two-mass distribution giving equal weights 0.5 to the one-point mass distribution at 0 and a *χ*^2^ with 1 df [28]. Practically, this means that standard 2-sided p-values have to be divided by 2.

### Case study on MOOD and depressive disorders

**Figure 8:**
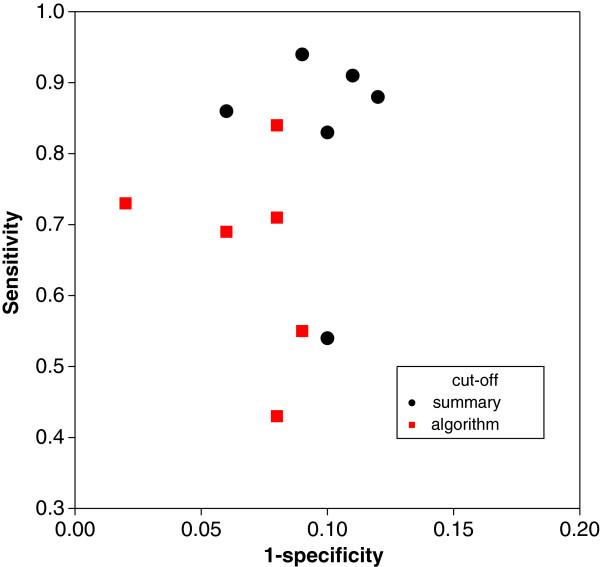
SROC diagram for the meta–analysis of MOOD and depression in patients in primary care.

The MOOD module of the Patient Health Questionnaire (PHQ-9) has been developed to screen and to diagnose patients in primary care with depressive disorders. The instrument consists of 9 questions, each scored from 0 to 3 points with a total score ranging from 0 to 27. In a meta–analysis of the diagnostic accuracy of MOOD, Wittkampf *et al.*[29] included 12 studies. These studies used either a cut-off of 10 (referred to here as “summary score”) or a more complex evaluation algorithm (“algorithm”). The complete data are listed in Table 5 and the associated SROC diagram is given in Figure 8. The impression from the graph is that the cut-off of 10 used by the summary score has a higher diagnostic accuracy than the alternative.

**Table 5:** Meta-Analysis of the diagnostic accuracy of the MOOD module and depression in patients in primary care as reference standard; TP = true positives, FN = false negatives, FP = false positives, TN = true negatives

**Study**	**Cut-off**	**TP**	**FN**	**FP**	**TN**
1	algorithm	65	26	104	1192
2	algorithm	70	13	74	846
3	sum score	62	10	27	429
4	sum score	36	5	65	474
5	sum score	55	11	43	392
6	algorithm	6	8	12	144
7	sum score	121	103	80	720
8	algorithm	11	5	5	76
9	algorithm	6	5	0	3
10	algorithm	85	31	9	460
11	sum score	15	1	4	42
12	sum score	96	10	23	187

The presence or absence of a cut-off value effect is now more formally investigated using a covariate cut-off, which is zero when the summary score with a cut-off value of 10 is used and one otherwise. The results are presented in Table 6. It can be seen that the covariate cut-off level “summary score” is associated with a higher diagnostic accuracy, although, as seen from the Wald statistics provided in column four of Table 6, the effect is not significant. We see a significant random effect (study; adjusted p-value 0.0274; see comment at the end of section “Case study on MMSE and dementia”), which indicates that the random study effect is needed in the analysis. It is not really surprising that the covariate cut-off is not significant, since the concept of the SROC is designed to accommodate the cut-off value variation. We will take up this point in the next section.

**Table 6:** Analysis of the cut-off effect for the meta-analysis of the MOOD module and depression in patients in primary care

**Effect**	**Parameter**	**SE**	**Z-value**
	**estimate**		
fixed			
Intercept	-2.5332	0.2817	-8.99
cut-off	0.4804	0.3966	1.21
random			
*τ*^2^ (study)	0.3239	0.1690	1.92

## Discussion

### Global versus local criteria

**Figure 9:**
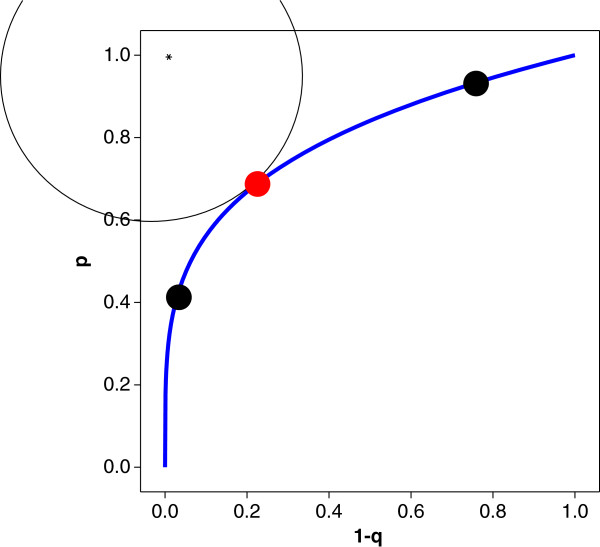
Different cut-off values with associated sensitivities and specificities on the same SROC curve with different Euclidean distances; the point on the circle has shortest Euclidean distance to the upper left vertex of the SROC diagram as indicated by the circle.

We have focussed on the PH measure so far, as it provides an appropriate measure for comparing SROC curves *globally*, in the sense that cut-off value variation will not necessarily effect the estimate of the SROC curve. The situation is illustrated in Figure 9.

Evidently, different cut-off values are associated with the same value of log*θ*, hence, the PH measure log*θ* is not the best measure to discriminate different cut-off values. This is not surprising, since the SROC curve is a concept designed for assessing the diagnostic accuracy of a diagnostic test globally, in the sense that it adjusts for different cut-off values. Hence, a measure that assesses local performance of the diagnostic is needed. Assuming that every cut-off value used in the meta–analysis is clinically meaningful, we suggest use of the (squared) Euclidean distance to the upper left corner (0,1) of the ROC diagram as a more meaningful measure to compare cut-off values:

(14) $${Ê}_{i}={\left(1-{\hat{p}}_{i}\right)}^{2}+{\left(1-{\hat{q}}_{i}\right)}^{2},$$

where ${\hat{p}}_{i}=y_{i}/m_{i}$ and ${\hat{q}}_{i}=x_{i}/n_{i}$. Each point in the SROC diagram has a unique circle with center (0,1) that passes through this point. In Figure 9, one such circle is illustrated which also has the smallest Euclidean distance among the three available points (since it has smallest radius among the three possible points with associated circles). In the following, we will consider the criterion (14) as an alternative criterion to choose the cut-off value.

Since we have changed the criterion, we need to determine the associated within-study variances. This can be accomplished easily, using the *δ*-method once more, to obtain

(15) $$\text{Var}(Ê)\approx 4{\left(1-\hat{p}\right)}^{2}\hat{p}(1-\hat{p})/m+4{\left(1-\hat{q}\right)}^{2}\hat{q}(1-\hat{q})/n,$$

where we have ignored study indexes for the the sake of simplicity. Using this criterion, we see in Figure 9 that cut-off values can vary considerably in their diagnostic accuracy, despite having identical diagnostic accuracy at a global level. We re-analyze the meta–analysis of MOOD and depression with respect to the (squared) Euclidean distance and provide the results in Table 7.

**Table 7:** Analysis of the cut-off effect for the meta-analysis of the MOOD module and depression in patients in primary care

**Criterion**	**Effect**	**Parameter**	**SE**	**Z-value**
	**estimate**			
PH measure	cut-off	0.4804	0.3966	1.21
Euclidean distance	cut-off	0.0563	0.0430	1.31

Evidently, both criteria lead to the same conclusion, namely that using the summary score with a cut-off value of 10 leads to the higher diagnostic accuracy (although the effect is not significant). It might also be worthwhile looking at the results of the likelihood ratio test: for the PH-measure as the outcome variable, the likelihood ratio test provides a value of 1.5; for the Euclidean distance, the value of the likelihood ratio test is 1.7, confirming the non-significance of the effect. Nevertheless, the analysis shows that the cut-off value of 10 provides the higher diagnsotic accuracy.

### Meta–analysis of magnetic resonance spectroscopy and prostate cancer

**Figure 10:**
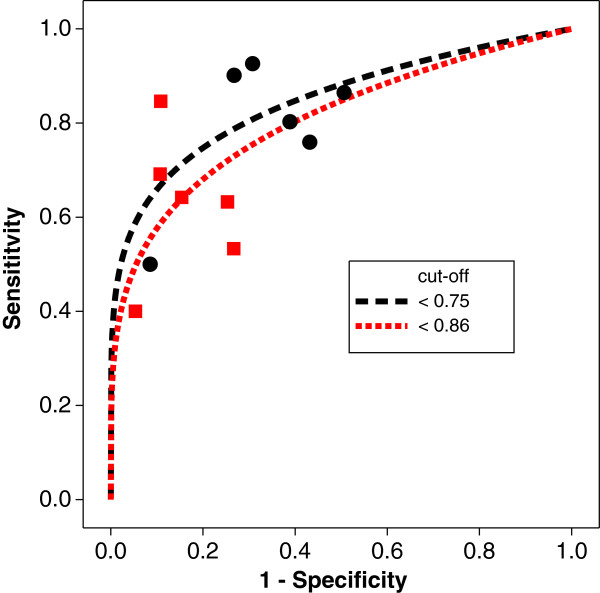
SROC diagram for the meta-analysis of the magnetic resonance spectroscopy and prostate cancer.

This case study provides an example where the use of a global or local criterion leads to a different conclusion. Magnetic resonance spectroscopy has the ability to discriminate between prostate cancer and benign prostatic hyperplasia, based on reduced citrate and elevated choline in the cancerous region. The diagnostic test works on a voxel of signal intensity ratios of (choline+creatine)/citrate. Two cut-off points are in use: <0.75 and <0.86. The results collected in a meta–analysis by Wang *et al.*[30] include 12 studies, as presented in Table 8; the associated SROC diagram is presented in Figure 10. From the graph, there is no obvious choice for the “best” cut-off value.

**Table 8:** Meta-analysis of the magnetic resonance spectroscopy and prostate cancer; TP = true positives, FN = false negatives, FP = false positives, TN = true negatives

**Study**	**Cut-off**	**TP**	**FN**	**FP**	**TN**
1	0.75	122	30	35	55
2	0.75	73	8	80	219
3	0.75	75	6	92	207
4	0.75	123	39	38	50
5	0.75	134	21	40	39
6	0.75	12	12	7	75
7	0.86	81	71	24	59
8	0.86	56	25	32	267
9	0.86	52	29	20	59
10	0.86	98	57	20	59
11	0.86	6	9	15	266
12	0.86	44	8	32	264

The fixed effects parts of the mixed model analysis, using the global PH measure and the local Euclidean measure as criteria, are presented in Table 9. It is interesting to note that the focus of the analysis, global or local, is an important part of the analysis. Globally, the better diagnostic accuracy is given by the cut-off value of 0.75, whereas better local performance is achieved with a cut-off value of 0.86, although neither analysis is significant.

**Table 9:** Analysis of the cut-off effect for the meta-analysis of the magnetic resonance spectroscopy and prostate cancer

**Criterion**	**Effect (reference)**	**Parameter**	**SE**	**Z-value**
		**estimate**		
PH measure	cut-off (<0.75)	0.2049	0.3516	0.58
Euclidean distance	cut-off (<0.75)	-0.0212	0.0573	-0.37

### PH measure and positive likelihood ratio

Another frequently used diagnostic measure is the positive likelihood ratio, defined as the ratio of sensitivity to false positive rate *P*(*T*=1|*D*=1)/*P*(*T*=1|*D*=0) or *p*/(1−*q*). It is different to the PH measure in that the ratio is taken on the log-scale: *θ*= log*p*/ log(1−*q*). Furthermore, if re-expressed as models, the positive-likelihood ratio corresponds to *p*=*θ*^′^(1−*q*), a straight line with no intercept, whereas the the PH measure corresponds to *p*=(1−*q*)^*θ*^, a straight line on the log-scale with no intercept. The positive likelihood ratio is a natural measure since it transfers the concept of relative risk (risk of a positive test in the diseased group to the risk of a positive test in the non-diseased group) to the diagnostic setting. However, it is less suitable as an (S)ROC model since it does not provide a function which connects the lower left vertex with the upper right vertex in the ROC diagram (which, in contrast, the PH-model does provide).

## Conclusions

The approach presented here is attractive since it is based on a simple measure of diagnostic accuracy per study, namely the ratio of log-sensitivity to log-false-positive rate. It also embeds the diagnostic meta-analysis problem into the well-known and much used mixed model setting. In particular, the analysis of effects of observed covariates on the diagnostic accuracy can easily be incorporated.

Controversies in the meta–analysis of diagnostic studies usually focus on comparability of studies. Study types might be case–control, cohort, cross–sectional or other. Studies might differ in the gold standard, severity of disease, or in the application of the diagnostic test. Patient populations might differ across studies, as might the cut-off value (defining positivity of the diagnostic test). All these different aspects, if observed, can be easily incorporated and analyzed as fixed effects in the special mixed model suggested here.

The occurrence of heterogeneity in the meta-analysis of diagnostic studies is more the rule than the exception; it is thus important to quantify the heterogeneity across studies due to the different sources. The approach provided here offers a more detailed investigation of heterogeneity according to the various observed sources and a residual heterogeneity (measured by *τ*^2^). This might allow us to construct a measure of relative residual heterogeneity, which might help to assess how trustworthy the results of a given meta-analysis may be. This will be investigated in future research.

In a recent study on the meta-analytical evaluation of coronary CT angiography studies, Schuetz *et al.*[31] investigated the problem of non-evaluable results that occur in the individual studies. They conclude that diagnostic accuracy measures change considerably depending on how non-evaluable results are treated. In fact, they conclude that 

parameters for diagnostic performance significantly decrease if non-evaluable results are included by a 3×2 table for analysis (intention to diagnose approach).

Twenty-six studies were included in their meta-analysis with a wide range of non-evaluable results from 0 to 43. Using the approach suggested here, it would be very easy to analyze the effect of non-evaluable results on the diagnostic accuracy by including the amount of non-evaluable results per study as a fixed effect in the proposed mixed model.

## Competing interests

The authors declare that they have no competing interests.

## Authors’ contributions

SC carried out all statistical and computing analysis. WB collected and prepared all meta-analytic data sets. HH conceived the theoretical modelling work and DB drafted the idea of the approach and finalized the manuscript. All authors read and approved the final manuscript.

## Pre-publication history

The pre-publication history for this paper can be accessed here:

http://www.biomedcentral.com/1471-2288/14/56/prepub
